# The Iranian female high school students' attitude towards people with HIV/AIDS: a cross-sectional study

**DOI:** 10.1186/1742-6405-5-15

**Published:** 2008-07-22

**Authors:** Kamyar Ghabili, Mohammadali M Shoja, Pooya Kamran

**Affiliations:** 1 Tuberculosis and Lung Disease Research Center, Tabriz University of Medical Sciences, Daneshgah St., Tabriz, Iran; 2 Students' Research Committee, Tabriz University of Medical Sciences, Attar Neishabouri Ave., Golgasht St., Tabriz, Iran; 3 Faculty of Medicine, Tabriz University of Medical Sciences, Daneshgah St., Tabriz, Iran

## Abstract

**Background:**

Acquired Immunodeficiency Syndrome (AIDS) has become an important public health hazard in Iran. It is believed that AIDS-related knowledge does not necessarily translate into behavior modification. Hence, it has been suggested that culturally appropriate educational campaigns should be implemented to obtain satisfactory outcomes. Here, we evaluated the female high school students' attitude towards HIV/AIDS in Tabriz, Iran to assess the cultural needs for the related educational programs and to discover sources of information about AIDS.

**Results:**

Anonymous, self-administered questionnaires were filled by the young female students. Among 300 students, 91% agreed that being an HIV carrier should not be an obstacle to obtaining education and employment. Moreover, 72.5% of the students declared that the community should be informed of HIV-positive people. In addition, one-tenth declared that they would feel extremely uncomfortable towards their HIV infected classmate. In addition, only 16% of the students stated that they would continue to shop at HIV infected grocer's store. The mass media and the experts were the major source and the most reliable source of information about AIDS, respectively.

**Conclusion:**

Tabrizian female students have overall negative attitudes towards HIV/AIDS. HIV/AIDS related educational campaigns should target the students, society and the families with emphasizing the leading roles of health staff.

## Background

Acquired Immunodeficiency Syndrome (AIDS) is among the most serious health problems of the 21^st ^century [1]. In Iran, the first case of Human Immunodeficiency Virus (HIV) infection was reported in 1987. This was followed by a rapid increase in the number of infected cases [2]. In 2004, there were officially 6532 Iranians living with HIV/AIDS, of which 95% were male [3]. This figure was increased to 16090 HIV positive individuals (94.6% male) in 2007. According to the recent report from Iran, 66.7% of AIDS patients are intravenous drug users, while 30% are infected by sexual contact [4]. Nevertheless, it is believed that these data underestimate the real number of HIV/AIDS cases [3]. Hence, the issue has become an important public health problem and several AIDS-related educational programs have been targeted on various populations including university and high school students [1,3].

Health education and prevention remain the main health care priorities in AIDS prevention [1]. It might be supposed that accurate knowledge of AIDS would reduce the risk behavior [1,2,5]. Nonetheless, numerous studies unveil that having adequate knowledge of HIV/AIDS does not necessarily translate into behavior modification [6-10]. Therefore, it has been suggested that culturally appropriate AIDS-related educational campaigns should be implemented to obtain satisfactory outcomes [9-11]. Moreover, the outcome of the educational campaigns focusing on the AIDS-related topics may be improved at schools if the level of the students' attitude is initially determined [1]. Although various studies have been performed worldwide to ascertain the students' knowledge and attitude towards HIV/AIDS, no comprehensive survey has been carried out among the Iranian students to assess their attitude towards this disease. Therefore, the current study aimed at evaluating the female high school students' attitude towards HIV/AIDS in Tabriz, Iran to assess the cultural needs for the related educational programs and to discover the sources of information about AIDS.

## Results

Three hundred female high school students from the fourth grade participated in the present study. The mean age of the respondents was 17.9 +/- 0.16 years (range 17–22). Two hundred and seventy three students (91%) agreed that being an HIV carrier should not be an obstacle to obtaining education and employment. The majority of the respondents (93%) believed that the community in which they were living was not protected from AIDS. Two hundred and sixty respondents (86.3%) recommended that special hospitals should be built for AIDS patients. Interestingly, 217 of the students (72.5%) declared that the community should be informed of HIV-positive people in order to strengthen the social awareness of potential source of the infection. Seventy seven students (25.5%) stated that they were not interested in having a friend who was an HIV carrier, whereas 83 respondents (27.5%) expressed that it would make no difference to have such a friend. Furthermore, only six students (2%) dissented from abortion of the HIV infected fetuses. Nearly one-tenth of the respondents disagreed with sending the HIV-positive children to special classes. A list of questions with the percentage of each response is provided in Table 1.

**Table 1:** The questionnaire and respondents' attitude towards HIV/AIDS (n = 300).

*Question*	*Totally agree (%)*	*Agree (%)*	*Neutral position (%)*	*Disagree (%)*	*Totally disagree (%)*
1. AIDS is the disease of the poor.	2	2	13.7	51	31.3
2. Most HIV-positive individuals are responsible for the acquisition of the infection.	13.7	29.4	5.9	35.3	15.7
3. Our community is protected from AIDS.	2	2	3	39.2	53.8
4. AIDS is a major hazard of the present time.	41.2	35.3	13.7	7.8	2
5. I will not be afflicted with HIV during my whole life.	24	34	20	20	2
6. HIV-positive people should be isolated from the general population.	14	12	12	40	22
7. Special hospitals should be created for AIDS patients.	56.9	29.4	9.8	2	2
8. AIDS patients should not receive education and employment.	1	2	6	41	50
9. The community should be informed of HIV-positive people.	31.3	41.2	11.8	9.8	5.9
10. Inefficacy of individuals leads them to acquire the AIDS.	9.8	13.7	23.5	37.3	15.7
11. I am not interested in having a friend who is a carrier of HIV.	25.5	23.5	27.5	17.6	5.9
12. AIDS sufferers are considered as victims of the social system.	13.7	43.1	23.5	15.7	3.9
13. AIDS patients should not take care of an orphan.	29.4	15.7	25.5	17.6	11.8
14. I would be responsible for caring an HIV-positive child.	4	20	42	16	18
15. The HIV tests should be voluntary and anonymous.	31.4	17.6	7.8	31.4	11.8
16. Most AIDS patients do not care if they infect other people too.	24	42	18	14	2
17. Pregnant women should be tested for the HIV.	60.8	33.3	3.9	1	1
18. Fetuses infected with HIV should be aborted.	60.8	21.6	13.7	2	2
19. Children who are HIV carriers should be sent to special schools/classes.	13.7	27.5	23.5	25.5	9.8
20. I feel sympathy towards AIDS patients.	8	28	46	10	8

The mean attitude score was 59.9 out of 100 (SD = 7.8, range 41 to 79). Considering a score of 75 as the cut-off point for a positive attitude, only 6% (n = 18) of the students had a positive attitude towards HIV/AIDS. Among the demographic items, educational level of father had a negative correlation with the attitude score (r = -0.131; p < 0.05). We found no significant correlation between the other demographic items (age, educational level of mother, family's mean monthly income) and the attitude score (p > 0.05).

In addition, the students were asked to suppose they found out that one of their classmates was suffering from AIDS. Only thirty respondents (10%) declared that they would feel extreme discomfort for such a situation. Interestingly, almost all the respondents (97.9%) stated that they would stay at the same school where the HIV-positive student was studying. Of these, one-fourth indicated that they would discontinue having any contact with their HIV-positive classmate, while more than half (52.1%) declared that they would treat their classmate in a usual manner. Two hundred and four students (68%) stated that they would feel discomfort to know that the neighborhood grocer had AIDS. Only 48 respondents (16%) stated that they would continue to shop at that grocery store.

The majority of the respondents (80%) indicated that the mass media (radio, television and newspapers) was the major source of their information about AIDS, followed by school educational materials (books and educational matters) (10%), and family and friends (10%). Furthermore, more than 75% of the students mentioned that they would rely on an expert's information on HIV/AIDS. In contrast, families and school teachers constituted the least reliable sources of AIDS-related information, respectively (3% and 2%).

## Discussion

In the present study, we surveyed a group of Iranian female high school students' attitude towards HIV-positive and AIDS patients. The overall findings from this study indicated a relatively negative attitude towards HIV/AIDS among Tabrizian female high school students. Similarly, a previous survey by Tavoosi and colleagues on a group of Iranian high school students in 2004 revealed an intolerant attitude towards AIDS and HIV positive patients [2]. Contrarily, Montazeri reported that Iranian people showed a more positive attitude towards HIV/AIDS than expected [3]. More than 40% of the students believed that HIV infected children should be sent to special schools/classes and not to regular classes. This is in agreement with those of Tavoosi et al. [2] and Brook [12]. In comparison, Pita-Fernández et al. [8] and Gañczak et al. [13] found the percentage of positive responses to a similar item as 5% and 73%, respectively.

In this study, the respondents also expressed affirmative attitudes towards some of the given items. In the current survey, only a few students thought that being a carrier of the HIV should be an obstacle to receiving education and employment. This is comparable to the study of Pita-Fernández and colleagues on a group of nurses in Spain [8]. Contrarily, in an earlier population-based study in Iran, Montazeri reported that more than a tenth of the respondents agreed that people with AIDS should not have right to study or work [3]. One-quarter of our respondents believed that HIV-positive people should be isolated from the general population. However, a recent report by Mazloomy and Baghianimoghadam indicates that more than 55% of the Iranian teachers agreed or strongly agreed with the similar statement [14].

The reports of rapid spread of HIV infection in different populations have no doubt increased the level of anxiety in the communities [2]. This may explain that why the majority of the surveyed students believed that their community was not protected from AIDS. This finding is similar to that of the survey by Tavoosi et al. on a group of Iranian high school students in the capital city of Tehran [2].

This study demonstrated that educational level of father was inversely associated with the level of attitude towards HIV/AIDS. The potential indications of this finding is unclear to us, however, the paternal factors affecting the attitudes of female students may demand further investigation. No significant correlation was found between age and the attitude score, well compared to the study by Montazeri [3]. However, this finding is in contrast with that of Mazloomy and Baghianimoghadam on a group of Iranian schoolteachers that indicated a negative correlation between age and the level of attitude [14].

The present study revealed that one-forth of the students would discontinue having any contact with their infected friend. In the study of Tavoosi et al., nearly one-third of students declared that they would avoid sitting near an infected student [2]. Surprisingly, in the study of Merakou et al. only 5% of Greek students declared that they would reject their infected friends [15]. Almost all the respondents of the current survey stated that they would stay at the same school where the HIV-positive student was studying. This is consistent with that of the similar study by Maswanya et al. indicating that 85% of the Japanese female college students would be able to study in the same class with HIV-positive classmates [16]. Moreover, in the present study, less than three quarters of the surveyed students felt discomfort about having an HIV-positive grocer in the neighborhood.

The mass media (radio, television and newspapers) were the most common means of obtaining information about HIV/AIDS. This finding is consistent with those of Brook [12], Tavoosi et al. [2] and Gañczak et al. [13]. However, physicians constituted the most reliable sources of information in our study. Hence, it seems that health professionals, in particular the physicians, should be involved in HIV/AIDS educational programs in the similar settings. Moreover, based on our results, the role of peer education programs in Iranian schools focusing on HIV/AIDS should be revised in order to set the most efficacious educations. Peer education programs have been started in Iranian guidance schools and high schools and thousands of students are being trained every year to educate their peers on HIV/AIDS. Consultants and health workers in guidance schools and high schools educate the selected students for efficient peer education [17].

## Conclusion

The overall findings from this study revealed an overall negative attitude about HIV/AIDS among Tabrizian female high school students. Based on the present study, one may recommend that the currently implemented HIV/AIDS related educational campaigns for the Iranian students should be extended beyond the schools to the society and families due to unsatisfactory role of families in AIDS education and prevention. Furthermore, the leading roles of health staff including physicians should be highlighted not only in HIV/AIDS educational programs at schools, but also in the media, which at present is the most frequent but not necessarily reliable source of information.

## Methods

This cross-sectional study was conducted in May 2005 in Tabriz, a city located in Northwest of Iran. The study population consisted of female high school students from the fourth grade who attended the educational institutes throughout Tabriz before entering the universities. Ten out of approximately 40 educational institutes in Tabriz were randomly selected. The data was collected by self-administered anonymous questionnaires. The questionnaire (Table 1) was developed based on a comprehensive literature review and consisted of four sections; (1) demographic items including age, educational level of both parents, and family's mean monthly income (4 items), (2) questions covering the students' attitudes towards HIV/AIDS (20 items), (3) questions regarding the students' probable behavior towards an HIV-positive person (5 items), and (4) multiple choice questions about the source of the students' information (2 items). A 5-point Likert scale ranging from 'totally agree' to 'totally disagree' was provided for each question. Responses ranged from 5 (total agreement) to 1 (total disagreement) for items 2, 12, 14, 15, 17 and 20; they ranged from 1 (total agreement) to 5 (total disagreement) for items 1, 3–11, 13, 16, 18 and 19. The maximum score from this questionnaire that reflects the most positive attitude is 100, and the minimum is 20, reflecting the most negative attitude. A panel of two epidemiologists and one statistician was invited to qualify and examine the questions. Data were presented in mean +/- SD or percentage, when appropriate. Statistical analysis was performed by using Statistical Package of Social Science (SPSS Inc., Chicago, IL) for Windows version 12.0. Spearman's correlation coefficient was calculated to study the correlation between quantitative variables (demographic items and total attitude score). In addition, permission to carry out the research was obtained from the directors of the institutes where the survey was performed. Students have been informed that their participation is voluntary and that the questionnaire is anonymous.

## Competing interests

The authors declare that they have no competing interests.

## Authors' contributions

KG participated in the design of study, acquisition of data, analysis and interpretation of data, drafting the article, and final approval of this version. MMS participated in the design of study, acquisition of data, interpretation of data, and drafting the article. PK participated in acquisition of data, analysis and interpretation of data, and drafting the article. All authors read and approved the final manuscript.
